# Vaccinia virus p37 interacts with host proteins associated with LE-derived transport vesicle biogenesis

**DOI:** 10.1186/1743-422X-6-44

**Published:** 2009-04-28

**Authors:** Yali Chen, Kady M Honeychurch, Guang Yang, Chelsea M Byrd, Chris Harver, Dennis E Hruby, Robert Jordan

**Affiliations:** 1 SIGA Technologies Inc, Corvallis, Oregon 97333, USA

## Abstract

**Background:**

Proteins associated with the late endosome (LE) appear to play a central role in the envelopment of a number of taxonomically diverse viruses. How viral proteins interact with LE-associated proteins to facilitate envelopment is not well understood. LE-derived transport vesicles form through the interaction of Rab9 GTPase with cargo proteins, and TIP47, a Rab9-specific effector protein. Vaccinia virus (VV) induces a wrapping complex derived from intracellular host membranes to envelope intracellular mature virus particles producing egress-competent forms of virus.

**Results:**

We show that VV p37 protein associates with TIP47-, Rab9-, and CI-MPR-containing membranes. Mutation of a di-aromatic motif in p37 blocks association with TIP47 and inhibits plaque formation. ST-246, a specific inhibitor of p37 function, inhibits these interactions and also blocks wrapped virus particle formation. Vaccinia virus expressing p37 variants with reduced ST-246 susceptibility associates with Rab9 and co-localizes with CI-MPR in the presence and absence of compound.

**Conclusion:**

These results suggest that p37 localizes to the LE and interacts with proteins associated with LE-derived transport vesicle biogenesis to facilitate assembly of extracellular forms of virus.

## Background

Vaccinia virus (VV) is the prototypical member of *Orthopoxviridae*, which replicates within the cytoplasm of permissive cells. At least four distinct types of enveloped infectious virus particles are produced during productive infection: mature virus, also referred to as intracellular mature virus or IMV, wrapped virus, also called intracellular enveloped virus (IEV), and two forms of extracellular virus (EV), cell-associated enveloped virus (CEV), and extracellular enveloped virus (EEV) [1,2]. IEV particles are precursors of extracellular forms of virus and are created by the wrapping of IMV particles in virus-modified membranes [3,4]. Electron microscopic evidence suggests that these membranes are derived from the TGN or post-TGN vesicles [3,5].

Envelopment of IMV particles requires the activity of p37, a highly conserved 37 kDa peripheral membrane protein encoded by the F13L gene [6]. Vaccinia p37 localizes to the *trans *Golgi, plasma and endosomal membranes and is shuttled between these various compartments through a clathrin-mediated endosomal pathway [7]. Golgi localization and intracellular trafficking of p37 requires palmitylation of two cysteine residues at positions 185 and 186 [8] as well as a putative phospholipase motif composed of histidine, lysine, aspartic acid (HKD) [5]. Targeted mutagenesis of these cysteine residues or amino acids within the HKD motif alters the intracellular distribution of p37 and inhibits IMV wrapping [9-12].

Vesicular transport and membrane trafficking are regulated by Rab proteins which are small (20–29 kDa) GTPases that belong to the Ras super family of proteins [13]. GTP-bound Rab proteins serve in cargo selection and act as scaffolds to nucleate vesicle assembly through interactions with cargo and rab-specific effector proteins [13]. A number of viruses that derive their envelopes from internal host membranes have been shown to interact with host components associated with the late endosome (LE) trafficking pathway [14-17]. The role of these virus-host interactions in the formation of the virus membrane is an area currently under investigation.

The LE is enriched for Rab9 protein that mediates recycling of cation-dependent mannose-6-phosphate receptor (CD-MPR) and cation-independent mannose-6-phosphate receptor (CI-MPR) from the LE to the *trans *Golgi network (TGN) [18]. Rab9-dependent recycling of CI-MPR is mediated through interactions with the tail interacting protein of 47 kDa (TIP47), a Rab9-specific effector [19,20]. TIP47 binds to a proline-rich motif found within the C-terminus of CI-MPR [18] and a diaromatic tyrosine-tryptophan (YW) motif found within the cytosolic tail of CD-MPR [21]. Likewise, TIP47 has been shown to interact with the HIV Env protein through a similar diaromatic YW motif [22], as well as the MA domain of the HIV Gag protein by way of a 9-residue region situated at the N-terminus [23]. RNAi-mediated depletion of Rab9 inhibited replication of human immunodeficiency virus type 1, filoviruses, and measles virus, which is consistent with Rab9-containing vesicles playing a role in virus assembly [16].

ST-246 is a small-molecule (MW = 376), potent pharmacological inhibitor of orthopoxvirus dissemination. Genetic resistance to ST-246 maps to the V061 gene product of cowpox virus, which is the homolog of VV p37 [24]. The small plaque phenotype observed in the presence of compound and the ability of the compound to prevent dissemination *in vivo *is consistent with the inhibition of extracellular virus formation. Thus, ST-246 is a useful tool for studying the mechanism of p37-dependent extracellular virus formation.

In this manuscript, plaque formation and wrapped virus formation were found to be dependent upon interaction of p37 with host LE-associated proteins in membrane fractions of infected cells. The association of p37 with Rab9 and CI-MPR but not, p230, a TGN-associated protein, could be inhibited by ST-246. Furthermore, mutation of a diaromatic amino acid motif in p37 reduced the interaction between p37 and TIP47 in membrane fractions from infected cell lysates, thereby preventing the formation wrapped virus particles. Taken together, these results suggest that p37 interacts with host proteins involved in LE trafficking to facilitate envelopment of IMV particles.

## Methods

### Cell culture

RK-13, BSC-40, BGMK, and Vero cells were obtained from American Type Culture Collection and maintained at 37°C and 5% CO_2 _in MEM medium (Invitrogen) supplemented with 10% fetal bovine serum (Invitrogen), 2 mM glutamine (Invitrogen) and 10 μg/ml gentamicin (Invitrogen).

### Antibodies

Immunofluorecent staining was performed using antibodies against GM130, p230 (BD Biosciences Pharmingen), and CI-MPR (Affinity BioReagents) at a concentration of 20 μg/ml. The secondary antibody was anti mouse-Alexa 594 (Molecular Probes) and was used at a concentration of 1 μg/ml. Antisera against viral proteins, B5 and L4, were generated at SIGA Technologies, Inc The primary antibodies used for immunoprecipitation and immunoblotting were: anti-CI-MPR (Affinity BioReagents), p230, polyclonal anti-GFP (Invitrogen), monoclonal anti-GFP (BD BioSciences) and polyclonal TIP47 (C-20) (Santa Cruz BioTechnology, Inc). Secondary antibodies included anti goat-HRP (Santa Cruz Biotechnologies), anti-mouse-HRP and anti-rabbit-HRP (Bio-Rad).

### Buoyant density centrifugation of radiolabeled virions

RK-13 cells were seeded in two 150 cm^2 ^diameter dishes at 1 × 10^7 ^cells per dish. The cultures were infected with 10 PFU/cell of vaccinia virus strain IHD-J in the absence or presence of 10 μM ST-246. At 3 hours post infection (hpi) the culture media was aspirated and replaced with thymidine-deficient MEM containing 12 μCi/ml of [methyl-^3^H]-thymidine (Amersham Biosciences GE Healthcare), either in the presence or absence of 10 μM ST-246. At 24 hpi, the culture supernatants were centrifuged at 4000 × g at 25°C for 5 min to remove cell debris. The supernatants were layered onto a 7-ml cushion of 36% sucrose in PBS and centrifuged at 40,000 × g at 4°C for 80 min. The pellet (extracellular virus) was resuspended in 1 ml PBS and stored on ice. BSC-40 cell monolayers were washed in 5 ml of PBS and harvested by scraping into 1 ml of hypotonic buffer (50 mM Tris pH 8.0, 10 mM KCl) and allowed to swell on ice for 10 min. The cells were subjected to two freeze-thaw cycles and then homogenized by 20 strokes in a Dounce homogenizer using a type-A pestle to release virus. The cellular debris was removed by centrifugation at 700 × g for 10 min at 4°C. The supernatants were layered on a 7-ml cushion of 36% sucrose in PBS and centrifuged at 40,000 × g for 80 min at 4°C. The pellet (cell-associated virus) was resuspended in 1 ml PBS and stored on ice. Both extracellular and cell-associated samples were layered on top of preformed CsCl gradients. The gradients were prepared in ultracentrifuge tubes by overlaying from bottom to top 3 ml of 1.30 g/ml, 4 ml of 1.25 g/ml and 5 ml of 1.20 g/ml CsCl solution, respectively. The samples were centrifuged at 100,000 × g for 3 hr at 15°C. Fractions (500 μL) were collected from the bottom of each gradient. Radiolabeled material from 20 μl of each fraction was quantified by liquid scintillation counting.

### Generation of recombinant VV

Marker rescue approach was used to generate vvF13L-GFP as previously described [24]. Briefly, a gel-purified PCR product of F13L-GFP containing 1 kb of flanking DNA was co-transfected with genomic DNA of VV strain WR using Lipofectamine 2000 (Invitrogen) according to the manufacturer's recommendations into BGMK cells infected with 2 PFU/cell of Shope fibroma virus 1 h prior to transfection. At 3 days post-transfection, progeny virus was plated onto BSC-40 cells and GFP-positive foci were isolated and subjected to three rounds of plaque purification. In addition, an F13L-deletion virus (vvΔF13LGFP) was created and purified as described above. In this recombinant mutant virus, the GFP open reading frame replaced the native F13L sequence following nucleotide 18.

### Plasmid construction and site-directed mutagenesis

A gel-purified PCR product of F13L-GFP containing 1 kb of flanking DNA (forward primer 5'-CATCCATCCAAATAACCCTAG-3'; reverse primer 5'-AGATACTCCTAGATACATACCATC-3') was TOPO cloned into pCR2.1 (Invitrogen) according to the manufacturer's instructions. Selected residues underwent targeted mutagenesis using the QuikChange^® ^Multi Site-Directed Mutagenesis Kit (Stratagene) according to the manufacturer's instructions. Mutagenesis primers were designed using the QuikChange^® ^Primer Design Program (Stratagene). Plasmid DNA was extracted using a QIAprep Spin Miniprep Kit (Qiagen). Residue changes were verified by DNA sequencing.

### *Trans *complementation assay

PCR products for transfection were prepared from each mutant plasmid using forward primer 5'-CTCTAATCGTGGAGATGATGATAGTTTAAGC-3' and reverse primer 5'-AGATACTCCTAGATACATACCATC-3'. PCR constructs were purified using the QIAquick PCR Purification Kit (Qiagen) and quantified by fluorometery. BSC-40 cells were seeded into 6-well tissue culture plates at a density of 3 × 10^5 ^cells/ml one day prior to transfection and then infected with 100 pfu/well of vvΔF13LGFP. At 1 hpi, the viral inoculum was removed and replaced with 1.5 ml of infection media. Transfections were carried out using 4 μg of purified PCR product and 10 μl of Lipofectamine 2000 (Invitrogen) according to the manufacturer's recommendations. Following an overnight incubation, the transfection inoculum was removed and replaced with 2.5 ml of infection media containing 1.5% methylcellulose. On day 3 post-infection, the plates were fixed with 5% glutaraldehyde in PBS and stained with 0.5% crystal violet containing 5% methanol.

### Subcellular fractionation

BSC-40 cells were infected with 5 PFU/cell of the wild type recombinant virus, vvF13LGFP. At 12 hpi, cells were washed once with PBS and harvested in 3 ml of HES buffer containing a protease inhibitor cocktail (1 mM EDTA, 250 mM sucrose, 20 mM HEPES, pH 7.4). The suspension was pelleted by centrifugation at 700 × g at 4°C for 5 min and the remaining volume was adjusted to 1.5 ml in HES buffer and passed 8 times through a 23 g needle followed by Dounce homogenization. After centrifugation at 800 × g for 5 min, the cleared cell lysate was centrifuged at 4,000 × g for 10 min to generate a low speed pellet and a low speed supernatant. The supernatant was cleared of mature virion particles by loading 1.7 ml onto a 36% sucrose cushion (Tris-HCl, pH 9.0) and subjected to centrifugation at 34,380 × g for 55 min at 4°C using a Beckman SW 60Ti rotor. The supernatant was collected and further centrifuged at 350,000 × g for 30 min to generate a high speed pellet enriched for LE and microsomes. The pelleted material was resuspended in 90 μl of HES buffer containing a protease inhibitor cocktail and divided in half. 45 μl was dedicated as the isolated membrane fraction and used as input controls. The remaining 45 μl was employed in immunoprecipitation procedures.

### Immunoprecipitation and immunoblotting

The protein content of each sample obtained from the membrane fractionation was quantified by a protein assay kit (Bio-Rad), adjusted to a concentration of 1 mg/ml and then preadsorbed with 25 μl of protein A-Sepharose 4B (50% slurry, Amersham Biosciences) at 4°C for 1 h to reduce non-specific protein-bead interactions. The samples were centrifuged at 500 × g for 15 s to remove the protein A-Sepharose 4B beads. Primary antibody was added to the supernatants to a final concentration of 2 μg/ml and incubated at 4°C for 12 h. Antibody-bound complexes were incubated with 40 μl of protein A-Sepharose (50% slurry) for 4°C for 2 h. Immunoprecipitates were collected by centrifugation at 500 × g for 15 sec and washed four times with buffer. Immunoprecipitates were resolved by 4–12% SDS-PAGE. Proteins were transferred to nitrocellulose membranes (Hybond-C, Amersham) and probed with specific antibodies to selected viral and cellular targets. Immune complexes were detected using ECL Western blotting detection kit (Amersham) according to the manufacturer's instructions and exposed to MR-type X-ray film (Kodak). Images for selected figures were collected and processed using Adobe Photoshop software followed by densitometry analysis using a BioRad Universal HoodII and Quantity I software package.

### Immunofluorescence analysis and confocal microscopy

Chamber slides (Fisher Scientific) containing BSC-40 cells were infected at 8 PFU per cell or transfected with 1 μg of plasmid DNA per well using Lipofectamine 2000 (Invitrogen) in the presence and absence of 10 μM ST-246. At 12–16 hpi, cells were washed in phosphate-buffered saline (PBS), fixed in 4% paraformaldehyde for 15 min at 25°C, rinsed with PBS, and permeabilized with 0.2% TritonX-100 for 5 min. Slides were blocked with 10% goat serum (Sigma Aldrich) in PBS for 30 min and then incubated for 1 h at 25°C with primary antibodies in goat serum-PBS. Cells were washed with PBS, incubated for 30 min at 25°C with secondary antibodies in goat-serum-PBS followed by three washes in PBS. The samples were mounted in ProLong Gold Antifade Reagent (Invitrogen Molecular Probes) containing DAPI to stain nuclear DNA. Analysis was performed using a Zeiss LSM 510 confocal laser-scanning microscope. Images were processed using LSM 510 Acquisition and Adobe Photoshop software.

### ST-246

ST-246 was synthesized at Pharmacore, (High Point, NC) and was dissolved in dimethyl sulfoxide (DMSO) (Sigma Aldrich) to a concentration of 10 mM. Where indicated, ST-246 was used at a concentration of 10 μM.

## Results and discussion

### ST-246 prevents the formation of wrapped viral particles

**Figure 1:**
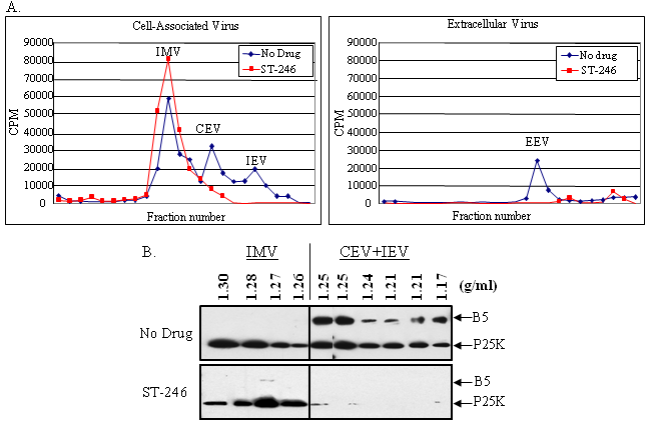
**Equilibrium centrifugation of radiolabeled virus particles in the presence and absence of ST-246**. Intracellular and extracellular virus particles were fractionated by equilibrium centrifugation on preformed cesium chloride gradients. (A) The radiolabeled viral DNA was measured by liquid scintillation counting. (B) The viral proteins in each fraction were detected by immunoblot analysis using antisera against L4 and B5 proteins. IMV, intracellular mature virus; CEV cell-associated enveloped virus; IEV, intracellular enveloped virus.

ST-246 targets VV p37 and has been shown to inhibit plaque formation *in vitro *and prevent systemic viral spread *in vivo *indicating that the compound inhibits the release of extracellular virus [24]. To determine if the block in EEV production was due to a lack of IEV production, VV was propagated in the presence and absence of ST-246, and virions were radiolabeled with tritiated thymidine and fractionated by equilibrium centrifugation (Fig. 1A). In the absence of compound, radiolabeled material from infected-cell lysates was partitioned into three distinct peaks of radioactivity, while radiolabeled material from the culture medium migrated as a single peak. Based upon the solution density of the peak fractions and immunoblot blot analysis, peaks from lysate samples were designated IMV, CEV and IEV, respectively, while the peak from the culture medium samples was designated EEV. In the presence of ST-246, only a single peak of radioactivity was observed from the cell associated virus fractions that migrated at the same density as IMV particles and no peak of radioactivity could be detected in virus from the culture medium. These results show that ST-246 inhibits IEV and CEV formation but not IMV formation during VV replication.

Immunoblot analysis of fractions from the equilibrium centrifugation experiment was conducted to confirm the identity of each type of viral particle in peak fractions. Immunoblots were probed with anti-L4 antiserum to detect viral cores and anti-B5 antiserum which is a component specific to wrapped viral particles (Fig. 1B). In the absence of ST-246, the L4 protein was detected in all fractions with a density between 1.168 g/ml and 1.300 g/ml, which is consistent with this protein being a component of all forms of VV particles. B5 protein was detected in fractions with a density between 1.168 g/ml and 1.250 g/ml, suggesting that these fractions contained IEV and CEV particles. In the presence of ST-246, L4 protein was detected in fractions with a density between 1.250 g/ml and 1.300 g/ml, while B5 protein was not detected in any of the fractions. Taken together, these results demonstrate that ST-246 inhibits wrapped virus formation and prevents extracellular virus production.

### p37 co-precipitates with TIP47 in infected cells

**Figure 2:**
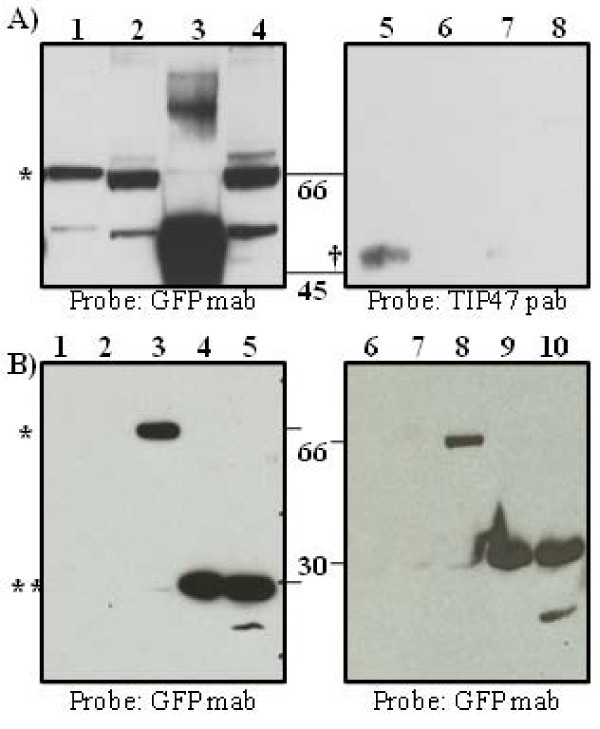
**p37-GFP and TIP47 co-immunoprecipitate in the presence of non-ionic detergent**. A. Membrane fractions from BSC-40 cells infected with vvF13LGFP at an MOI of 5 were extracted as described in Materials and Methods. Protein extracts obtained from the high speed pellet were resuspended in a buffer containing a non-ionic detergent (0.5% NP-40, 150 mM NaCl, 20 mM Tris, pH7.4) and immunoprecipitated with antibodies specific for TIP47 or GFP followed by SDS-PAGE analysis and immunoblot. Antibody raised against the trans Golgi marker, TGN46 (Sigma Aldrich) was used as a non-specific control for immunoprecipitation. Lane 1, IP using TIP47 pab; Lane 2, Unbound Fraction; Lane 3, IP using TGN46 pab; Lane 4, Unbound Fraction; Lane 5, IP using GFP pab, Lane 6, Unbound Fraction, Lane 7, IP using TGN46 pab; Lane 8, Unbound Fraction; B. Immunoblot of whole cell extracts and membrane fractions immunoprecipitated with GFP pab from BSC-40 cells mock-infected or infected with vvWR, vvGFP, vvΔF13LGFP, or vvF13LGFP at an MOI of 5 probed with GFP mab. Lane 1 and 6, mock-infected, Lane 2 and 7, vvWR, Lane 3 and 8, vvF13LGFP, Lane 4 and 9, vvΔF13LGFP, Lane 5 and 10, vvGFP. IP, immunoprecipitated; pab, polyclonal antibody; mab, monoclonal antibody; ** pGFP; * p37-GFP fusion protein; † TIP47 protein.

Plaque formation requires p37 activity that facilitates wrapping of IMV in virus-modified membranes derived from a post TGN-compartment to produce egress competent viral particles. A conserved YW motif (positions 253 and 254) was identified in the p37 protein that was identical to a motif found in HIV Env, the same motif shown to mediate the association of Env with TIP47 [22]. In order to establish whether or not p37 physically interacts with TIP47, virion-free membrane fractions enriched for LE-proteins were isolated from BSC-40 cells infected with vvF13LGFP as described in the Material and Methods and resuspended in a buffered solution containing a non-ionic detergent. The fractions were then subjected to immunoprecipitation using polyclonal antibodies to either TIP47 (Fig. 2A, lanes 1 and 2), GFP (Fig. 2A, lanes 5 and 6), or TGN46 as a negative control (Fig. 2A, lanes 3, 4, 7, and 8). The immunoprecipitated proteins (Fig. 2A, lanes 1, 3, 5 and 7) or supernatants (unbound fraction) from the immunoprecipitation reaction mixtures (Fig. 2A, lanes 2, 4, 6 and 8) were resolved by SDS-PAGE, transferred to a nitrocellulose membrane and probed with either a monoclonal antibody to the GFP portion of the p37-GFP fusion protein (left panel) or a polyclonal antibody to TIP47 (right panel). p37-GFP co-precipitated with TIP47 (lane 1) and was also detected in the unbound fraction (lane 2). Likewise, TIP47 co-precipitated with p37-GFP (lane 5), however, TIP47 was not detected in the unbound fraction (lane 6) most likely because TIP47 is primarily a soluble cytosolic protein and would not normally copurify with membrane fractions unless tethered to a specific membrane associated cargo protein. A non-specific band of approximately 55 kDa was also detected in the blot probed with antibody to GFP and likely represents the heavy chain portion of the TGN46 antibody employed in the immunprecipitation reaction (Fig. 2, lane 3). p37 was found in the unbound fraction (Fig. 2, lane 4) when polyclonal antibody specific for TGN46 was used in the immunoprecipitation reactions while TIP47 could not be detected in the bound (Fig. 2, lane 7) or unbound fractions (Fig. 2 lane 8). Taken together, these results suggest that a physical interaction exists between p37 and TIP47. While interaction of membrane proteins in the presence of non-ionic detergent suggests direct physical interaction, additional experiments are required to prove specificity of this interaction.

To demonstrate antibody specificity, total cell lysates were prepared from BSC40 cells that were either mock-infected or infected with vvWR, vvGFP, vvΔF13LGFP, or vvF13LGFP. A portion of the lysate was immunoprecipitated with a polyclonal antibody to GFP. Immunoblots of the lysate material pre- and post-immunoprecipitation were probed with a monoclonal antibody to GFP (Fig. 2B). A band of approximately 66 kDa was observed on the immunoblots in samples (Fig. 2B, lane 3 and 8) infected with vvF13L-GFP migrating at the predicted size of the p37-GFP fusion protein. A band of approximately 27 kDa was observed in samples (Fig. 2B lane 4,5,9, and 10) infected with vvGFP and vvΔF13LGFP corresponding to the size of GFP. No bands were observed in mock-infected samples (Fig. 2B lanes 1 and 6) or samples infected with vvWR (Fig. 2B, lanes 2 and 7). These results suggest that the both monoclonal and polyclonal antibodies specific for GFP do not cross react with other viral and cellular proteins.

### A conserved YW motif within p37 is required for complimentation of an F13L-deletion virus

**Figure 3:**
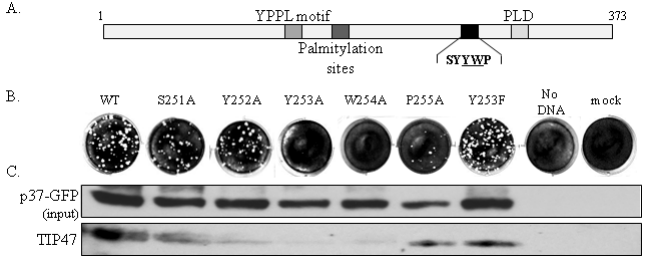
**Mutation of the YW motif in F13L blocks association of p37-GFP with TIP47 and prevents complementation of an F13L-deleted virus**. (A) Diagram of the VV F13L gene with functional domains indicated. (B) Complementation experiment demonstrating a panel of mutant F13L alleles containing the indicated A or F substitutions. The F13L allelescontained a GFP tag at the C-terminus to facilitate immunoprecipitation of p37. PCR products containing the indicated F13L allele were transfected into BSC-40 cells infected with an F13L-deletion virus. At 3 days post infection/transfection, plaques were visualized by staining with crystal violet, (C) Membrane fractions, prepared as described in Materials and Methods, from infected and transfected cells were immunoprecipitated in hypotonic buffer with anti-GFP antibody and probed with TIP47 antibody (lower panel). The upper panel shows the input controls.

To determine whether the YW motif in p37 (Fig. 3A) is required for interaction with TIP47, PCR-generated DNA fragments of F13L containing alanine substitutions within the YW motif were used to complement plaque formation of an F13L-deleted virus. Co-immunoprecipitation was then conducted to assess the ability of p37 expressed from the mutated F13L alleles to associate with TIP47 in virion-free membrane fractions obtained from infected cells. Changing either Y or W to A at positions 253 and 254, respectively, or both residues simultaneously (data not shown) prevented co-immunoprecipitation of p37 with TIP47 (Fig. 3C). In contrast, changing Y at position 253 to phenylalanine to maintain the aromatic character of the motif, or changing amino acids surrounding the YW motif to A (residues 251, 252, and 255 – Fig. 3A) reduced the association of p37 with TIP47 to varying degrees (Fig. 3C). Introduction of these mutations did not alter the subcellular localization of F13L in infected cells (see Additional File 1).

VV recombinants containing deletions in F13L produced normal levels of IMV particles but failed to form plaques within a 3-day incubation period at 37°C. After a 7-day incubation period plaques generated by F13L-deleted recombinants were comparable in size to plaques generated by wild-type virus after a 1-day incubation period. This strong plaquing phenotype was used to measure the ability of mutated F13L alleles to complement plaque formation of an F13L-deleted virus. Consistent with the co-immunoprecipitation data presented in Fig. 3C, p37 variants containing alanine substitutions at either Y253 or W254 respectively, were unable to complement plaque formation of an F13L-deleted virus (Fig. 3B). Alanine substitutions at positions surrounding the YW motif did not block complementation of the F13L-deleted virus (Fig. 3B) suggesting that the YW motif and not amino acids surrounding this motif are required for extra cellular virus formation. Complementing plaque formation by transfecting PCR products encoding p37 could occur through recombination of the F13L alleles into the viral genome or by transient expression of active p37. This distinction, however, does not affect the interpretation of the experiment. Taken together, these observations suggest that the aromatic nature of the YW motif is required for plaque formation and that the YW motif contributes to the interaction of p37 with TIP47 in membrane fractions of infected cells.

Confocal laser scanning microscopy was conducted to rule out the possibility that the p37 expressed from the Y253A and W254A mutants was mislocalized and no longer capable of complementing plaque formation of the F13L-deleted virus. BSC-40 cells were infected with VV, strain WR, at an MOI of 1 and then transfected with 200 ng of PCR-generated DNA encoding wild-type or mutant p37-GFP alleles. Approximately 50 to 100 infected and transfected cells per mutant construct were evaluated for altered intracellular localization of p37-GFP. The fluorescence profile obtained from the p37-GFP fusion protein was evaluated against a TGN marker and found to be indistinguishable from wild type p37 suggesting that alanine substitution in and around the YW motif does not alter the sub-cellular localization of p37 (Fig. S1).

### ST-246 inhibits association of p37-GFP with LE proteins in membrane fractions from infected cells

**Figure 4:**
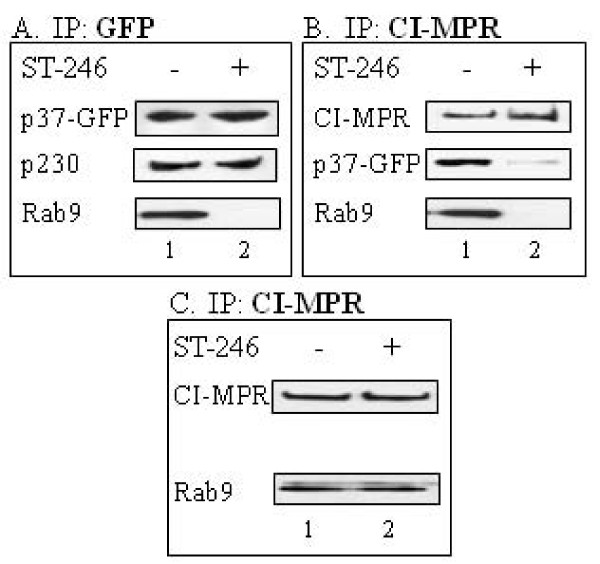
**ST-246 affects the co-immunoprecipitation of membrane-associated p37-GFP, Rab9 and CI-MPR but not p230**. BSC-40 cells were infected (A and B) with 5 PFU per cell of vvF13LGFP or mock-infected (C) in the absence (column 1) or presence (column 2) of 10 μM ST-246. The membrane fractions were extracted as described in Materials and Methods, resuspended in hypotonic buffer and immunoprecipitated as indicated. Blots were probed with (A) p230 or Rab9 antibody, (B) GFP or Rab9 antibody, and (C) Rab9 antibody. The top row for each are input controls and are probed as indicated.

Based on the observations that the interaction of p37 with TIP47 may be involved in plaque formation and that ST-246, which targets p37 activity, inhibited wrapped virus formation, the potential association of p37 with host proteins known to be involved in Rab9-dependent vesicle formation was evaluated. BSC-40 cells were mock-infected or infected in the presence or absence of ST-246 with vvF13LGFP, a recombinant virus that expresses a p37-GFP fusion protein. The isolated membrane fraction was immunoprecipitated in hypotonic buffer with anti-GFP to pull out the p37-GFP-containing complexes. Immunoblot analysis of the precipitated proteins using antibodies specific for Rab9 or the TGN resident protein, p230, was used to measure the association of these proteins with p37. A similar experiment was performed to measure the association of CI-MPR with p37-GFP and Rab9. In the absence of ST-246, immunoprecipitation of p37-GFP co-precipitated p230 and Rab9 protein (Fig. 4A, Lane 1). Likewise, immunoprecipitation of CI-MPR co-precipitated p37-GFP and Rab9 (Fig. 4B, Lane 1). In the presence of ST-246, the amount of p230 co-precipitating with p37-GFP remained constant while the amount of Rab9 associated with p37-GFP was undetectable (Fig. 4A, Lane 2). Similarly, the amount of both p37-GFP and Rab9 co-precipitating with CI-MPR was reduced in cells treated with ST-246 (Fig. 4B, Lane 2). ST-246 did not affect the co-precipitation of Rab9 with CI-MPR in samples from mock-infected cells (Fig. 4C, Lanes 1 and 2). Taken together, these results imply that ST-246 inhibits the interaction of p37-GFP with cellular proteins associated with Rab9-dependent vesicle formation but not proteins associated with the TGN in membrane fractions from infected cells. Moreover, ST-246 does not alter the association of CI-MPR with Rab9 in uninfected cells suggesting that the inhibitory effects of ST-246 are virus-specific and not the result of an interference with Rab9-dependent transport processes in uninfected cells.

### ST-246 inhibits the co-localization of p37-GFP with LE proteins in the context of viral infection

**Figure 5:**
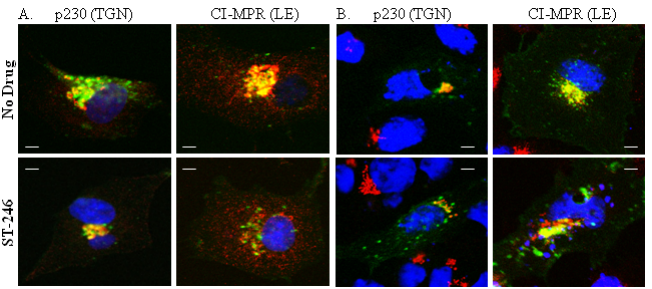
**Co-localization of p37-GFP with p230 and CI-MPR in infected and uninfected cells in the presence and absence of ST-246**. Cell monolayers were grown on chamber slides and (A) infected with 8 PFU/cell of vvF13LGFP or (B) transfected with 1 μg of plasmid expressing p37-GFP in the presence or absence of 10 μM ST-246. At 14–16 hpi cells were fixed in 4% paraformaldehyde, permeablized with 0.2% TritonX-100 and stained for 1 hour with anti-p230 antibody or anti-CI-MPR antibody. Proteins were visualized using Alexa 594-conjugated secondary antibody. Samples were mounted in ProLong Gold Antifade Reagent (Invitrogen Molecular Probes) containing DAPI for nuclear staining and analyzed using a Zeiss LSM 510 confocal laser-scanning microscope. Images are representative of 2 separate experiments in which a minimum of 50 infected cells displaying a similar fluorescent phenotype were observed. Images were collected and processed using LSM 510 acquisition and Adobe Photoshop software. Bar, 5 μm.

To support the results of the co-immunoprecipitation experiments, BSC-40 cells were infected with vvF13LGFP in the presence and absence of ST-246 and the subcellular localization of p37-GFP protein was determined by immunofluorescence analysis and confocal microscopy. The results of these studies are presented in Fig. 5 and consist of images that are representative of a minimum of 50 infected cells. In the absence of ST-246, a discrete distribution of p37-GFP was observed, with no signal in the nucleus (Fig. 5A). This signal co-localized with cellular markers specific for GM130 (cis-Golgi) [25], p230 (TGN)[26], and CI-MPR (LE) [27] as shown by the pattern of yellow in the merged images (Fig. 5 and data not shown). These observations are in agreement with previous reports by other groups [3,5]. In the presence of ST-246, however, p37-GFP co-localized with p230, with very little, if any, co-localization with CI-MPR. Consistent with the co-immunoprecipitation studies (Fig. 4), these observations suggest that ST-246 inhibits the intracellular trafficking of the p37 protein to LE compartments in infected cells. Thin-slice analysis (Z-stacks) by confocal microscopy further supported the above observation (data not shown).

Since the effect of ST-246 on co-localization of p37-GFP with LE proteins was observed in the context of virus infection, it was important to determine whether this effect required other viral proteins or was specific for p37. Vero cells were transfected with the plasmid pSI-F13L-GFP expressing p37-GFP in the presence or absence of ST-246 and analyzed by immunofluorescence and confocal microscopy (Fig. 5B). The pattern of p37-GFP signal was not affected by the presence of ST-246 and p37-GFP appeared to co-localize with p230 and CI-MPR in the presence and absence of ST-246 as measured by the intensity of the yellow color of the merged images. These results suggest that ST-246 inhibits a virus-specific activity required for co-localization of p37 with LE proteins and is consistent with the observation that functional p37 is required for co-localization of the EEV-specific envelope protein, B5, to post-Golgi vesicles [11].

### ST-246 reduces p37-GFP association with vaccinia virus B5 protein and LE proteins in membrane fractions from infected cells

**Figure 6:**
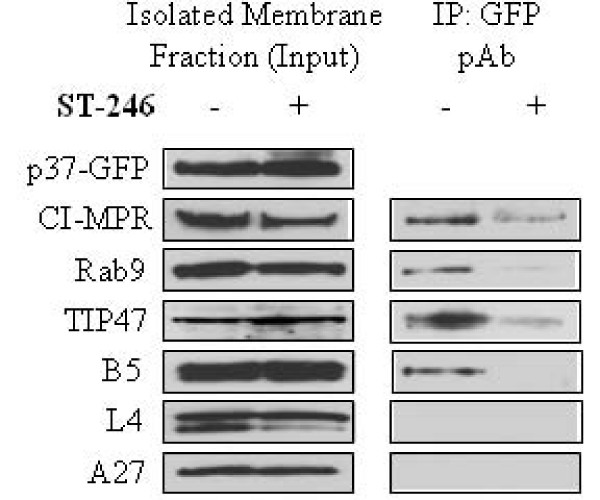
**ST-246 inhibits membrane precursor biogenesis. BSC-40 cells were infected with 5 PFU per cell of vvF13LGFP in the presence or absence of 10 μM ST-246**. Membrane extracts were prepared as described in Materials and Methods and analyzed by immunoblot (left panel). The remaining supernatant was then subjected to immunoprecipitation (right panel). Blots were probed with the indicated antibodies. Left panel, input controls; right panel, immunoprecipitated samples.

The co-localization of p37 with B5 protein in infected cells is thought to be an indicator of envelope precursor formation [11]. To examine whether p37-GFP co-fractionated with B5 and LE marker proteins, isolated membrane fractions were subjected to co-immunoprecipitation in hypotonic buffer with anti-GFP polyclonal antibody. Immunoblot analysis of the isolated membrane fraction prior to immunoprecipitation (input) showed the presence of the host factors CI-MPR, Rab9 and TIP47 as well as the viral IEV component, B5 (Fig. 6, left panel), in the presence and absence of ST-246. Immunoprecipitation of the membrane fraction using antibodies to p37-GFP in the absence of ST-246 co-precipitated CI-MPR, Rab9, TIP47 and B5 (Fig. 6, right panel). In the presence of ST-246, the amount of CI-MPR, Rab9 and TIP47 co-precipitating with p37-GFP was greatly reduced and B5 was undetectable. Although the membrane fractions were cleared of intact virion particles by velocity sedimentation as described the Materials and Methods, it is likely that other viral components, particularly structural proteins, which tend to be highly expressed, are present in the membrane fraction but not associated with the p37-containing membranes. To investigate this possibility, the isolated membrane fraction was probed with antibodies specific for the viral core proteins L4 and A27 both pre- and post- immunoprecipitation. The A27 and L4 proteins were detected in the input material (Fig. 6, left panel) in the presence and absence of ST-246 but not in the complex immunoprecipitated with antibody to p37-GFP (Fig. 6, right panel). This result suggests that these fractions contain envelope precursors and viral core proteins, but not intact IEV particles. These results suggest association of LE proteins with p37- and B5-containing membrane fractions is sensitive to inhibition by ST-246.

### p37 expressed from an ST-246-resistant VV variant interacts with Rab9 and B5 in the presence and absence of compound

**Figure 7:**
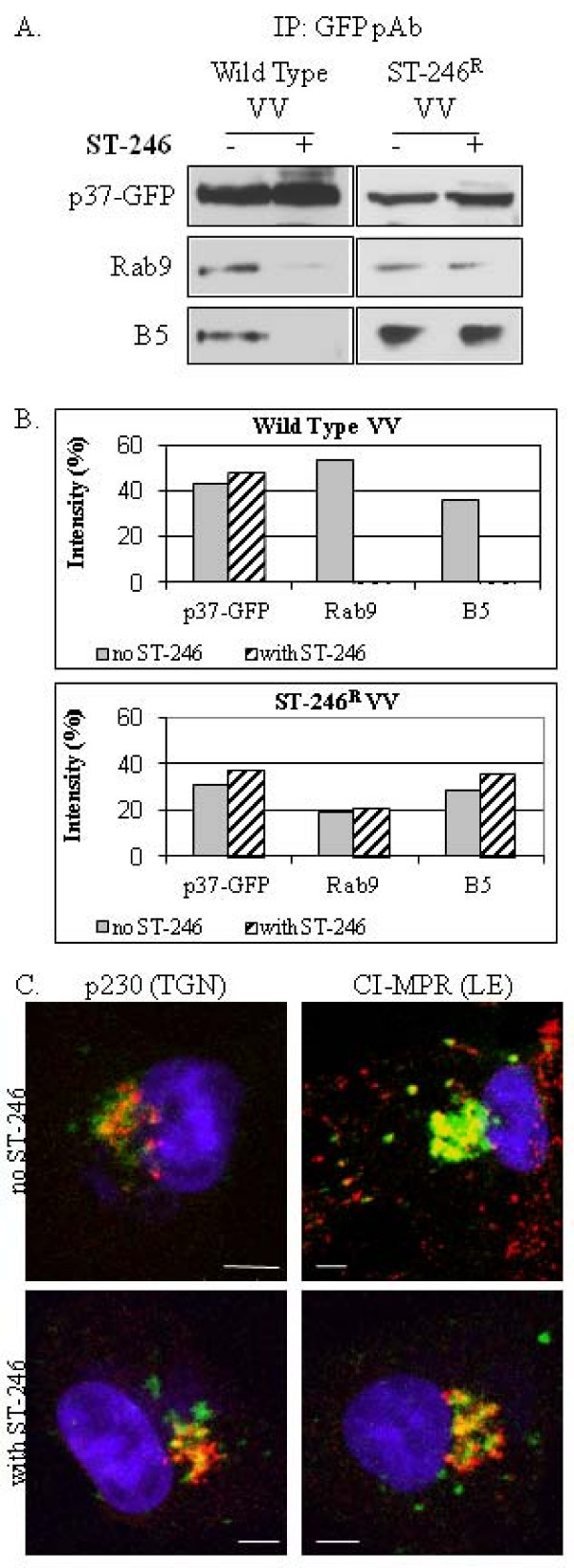
**Co-immunoprecipitation and subcellular localization of Rab9 and B5 protein with p37-GFP expressed from vvF13LGFP or an ST-246 resistant VV virus variant**. (A) BSC-40 cells were infected with wild type VV (left) or an ST-246-resistant variant that exhibits reduced susceptibility to ST-246 (right) in the presence or absence of 10 μM compound. The membrane fractions were extracted as described in Materials and Methods, resuspended in hypotonic buffer and immunoprecipitated with anti-GFP polyclonal antibody. Blots were probed with Rab9 antibody or antiserum to B5 as indicated. Top row: input controls; (B) Quantitative comparison of immunoblot intensity in the absence and presence of ST-246; (C) Cell monolayers were grown on chamber slides and infected with 8 PFU/cell of an ST-246-resistant variant in the presence or absence of 10 μM ST-246. At 14–16 hpi cells were fixed in 4% paraformaldehyde, permeablized with 0.2% TritonX-100 and stained for 1 hour with anti-p230 antibody or anti-CI-MPR antibody. Proteins were visualized using Alexa 594-conjugated secondary antibody. Samples were mounted in ProLong Gold Antifade Reagent (Invitrogen Molecular Probes) containing DAPI for nuclear staining and analyzed using a Zeiss LSM 510 confocal laser-scanning microscope. Images are representative of experiments in which a minimum of 50 infected cells displaying a similar fluorescent phenotype were observed. Images were collected and processed using LSM 510 acquisition and Adobe Photoshop software. Bar, 5 μm.

To confirm that inhibition of the association of LE proteins with p37-containing membrane fractions by ST-246 is mediated through p37, BSC-40 cells were infected with wild type vvF13LGFP or an ST-246 resistant (ST-246^R^) variant, which contains an Asp to Tyr change within the p37 protein. This amino acid change increases the concentration of compound required to inhibit 50% of the virus-induced cytopathic effects (EC_50_) by more than 1000-fold. The EC_50 _of ST-246 for wild-type VV is 50 nM while the EC_50 _for the ST-246^R ^variant is > 50 μM. Immunoblot analysis of proteins immunoprecipitated with anti-GFP polyclonal antibody from membrane fraction from wild type virus-infected cells showed an association of p37 with Rab9 and B5 in the absence of ST-246. In the presence of ST-246, these interactions were nearly undetectable (Fig. 7A, left). In contrast, immunoblot analysis of immunoprecipitated proteins from membrane fractions of ST-246^R ^variant virus-infected cells showed an association of Rab9 and B5 with p37 in the presence and absence of ST-246 (Fig. 7A, right). Densitometric analysis of the immunoblots presented in Fig. 7A shows that the amount of Rab9 and B5 that co-precipitate with p37-GFP in the presence of ST-246 was greatly reduced in wild-type VV-infected cells, compared to ST-246^R ^virus-infected cells (Fig. 7B).

To support the co-immunoprecipitation studies described above, the intracellular localization of p37, p230, and CI-MPR within cells infected by the ST-246^R ^variant in the presence and absence of ST-246 was examined by confocal microscopy (Fig. 7C). The images presented are representative of 50–100 infected cells throughout the entire sample and were confirmed by thin-slice (Z-stack) analysis. As observed in previous experiments (Fig. 4 and data not shown), p37 co-localized with p230 in the presence and absence of ST-246 (indicated by the pattern of yellow in the merged images) (Fig. 7C, left). Moreover, unlike p37 expressed from wild type vaccinia virus infected cells, the p37 expressed from the ST-246^R ^variant co-localized with CI-MPR in the presence and absence of ST-246 (Fig. 7C, right). Taken together, these results suggest that ST-246 inhibits the formation of a membrane complex containing p37 and LE proteins.

## Discussion

A prerequisite for production of extracellular forms of VV is the assembly of IEV particles that involves wrapping of IMV particles in virus modified membranes derived from the host. Induction of this so-called wrapping complex, also described as an envelope precursor membrane or a post-TGN-vesicle, requires activities associated with the p37 protein [28]. The wrapping complex incorporates viral and cellular proteins that would normally co-localize to the Golgi and LE and is also enriched for selected viral outer envelope proteins [1,5]. The wrapping complex is thought to be the biologically active entity that enwraps IMV particles to produce IEV particles [5,11]. In this manuscript, we show that induction of a membrane complex containing viral p37 and B5 proteins and host LE proteins involved in transport vesicle biogenesis requires interaction of p37 with LE proteins and can be inhibited by ST-246, a specific orthopoxvirus egress inhibitor.

The LE has been implicated as a potential source of membranes for the formation of wrapped virus [4,11]. These observations led us to focus on the involvement of proteins associated with the LE in extracellular virus production. Rab9 and TIP47 are both enriched in the LE and form a scaffold for transport vesicle formation that shuttles CI-MPR from the LE to the Golgi. Immunoprecipitation of p37-containing membrane fractions co-precipitated TIP47 and Rab9. Targeted alanine (A) substitutions of a conserved diaromatic (YW) motif in p37, the same motif that has been shown to mediate the interaction of TIP47 with HIV Env [22], blocked the association of p37 with TIP47 in membrane fractions from infected cells. Furthermore, A substitutions within the YW motif that reduced interactions with TIP47 also failed to complement plaque formation of an F13L-deleted virus. Maintaining the diaromatic nature of the YW motif retained the ability of TIP47 to associate with p37 and rescue the small plaque phenotype of the F13L-deleted virus suggesting that the diaromatic nature of this motif is necessary for extracellular virus formation. In addition, ST-246 specifically blocked association of p37 with Rab9, TIP47, and CI-MPR-containing membranes as well as with the VV B5 protein but not p230, a TGN marker protein, in the context of viral infection. These data support a model whereby p37-containing membranes associate with host proteins required for induction of LE transport vesicles to facilitate EV particle production.

The observation that the interaction between Rab9 and CI-MPR appeared to be ST-246-sensitive only in the context of viral infection suggests that Rab9-mediated transport pathways are occupied by p37-dependent virus-specific processes. Viral p37 is expressed at high levels and represents a major source of cargo within the infected cell, potentially sequestering available Rab9-containing membranes to form the wrapping complex. By disrupting p37-Rab9 interactions, ST-246 blocks the formation of a membrane complex involved in EV biogenesis and perhaps prevents Rab9-dependent vesicle formation and association of cellular cargo, such as CI-MPR, with Rab9-containing membranes. While this phenomenon remains under investigation, it exemplifies the high degree of specificity of ST-246 for infected cells and may explain the observation that ST-246 has little or no toxicity in cell culture and in animals.

The origin of the virus modified membranes that form the wrapping complex has been controversial. Both the TGN and endosomes have been implicated as the site of IMV wrapping [3,4,11,28]. Immuno-EM studies have shown that the virus-modified membrane system, which appears as a double membrane structure 'modified' with viral membrane proteins, could be stained with lectins specific for glycoproteins that have been processed in the TGN but not the *cis *or medial Golgi [3]. Moreover, Rab5 and Rab7, proteins normally associated with early and late endosomes, respectively, were not found associated with wrapping membranes [3]. These studies also show that endocytic tracers (HRP and BSA-gold) were found in the lumen of the wrapping cisternae. In addition, a number of IMV particles could be found associated with endosomes. Our data is consistent with these observations and suggests that the virus modified membranes involved in EV formation are derived from the late endosome and are induced by interaction of p37 with LE proteins involved in transport vesicle biogenesis.

Rab proteins are required for transport vesicle biogenesis and are essential components of the membrane trafficking system in the cell. Rab9 is predominantly localized to the LE compartment and plays a role in cargo selection and transport vesicle formation through interactions with TIP47 [19,20]. RNAi-mediated disruption of this pathway reduces replication of human immunodeficiency virus, filoviruses, and measles virus suggesting a common theme among diverse virus families that acquire their envelope from this subcellular compartment [16]. Although RNAi-mediated knock-down of Rab9 or expression of dominant-negative mouse-specific Rab9 allele did not inhibit plaque formation of VV, strain IHDJ (data not shown), the association of p37 with LE components correlates with induction of a p37-containing membrane complex containing LE proteins and efficient plaque formation. The inability to block plaque formation by interfering with Rab9 expression or activity could be due to residual Rab9 activity remaining after RNAi knock-down or species incompatibility between dominant-negative mouse Rab9 alleles and primate-specific host components involved in membrane vesicle formation. Alternatively, vaccinia virus may be less discriminating in the use of Rab proteins and other host components involved in wrapping complex formation. Indeed, vaccinia virus can use multiple host kinases to facilitate release of cell-associated extracellular virus into the extracellular space providing precedent for this idea [29].

## Conclusion

The p37 protein may participate directly in the biogenesis of the wrapping complex through a conserved histidine-lysine-aspartate (HKD) phospholipase motif that has been shown to be essential for IMV wrapping. Targeted mutagenesis of this motif or addition of a specific phospholipase D inhibitor, reduced IMV wrapping and inhibited extracellular particle formation [5,11]. This result suggests that phospholipase activity is important for p37 function. Indeed, purified p37 has been shown to have broad-spectrum lipase activity [30]. The role of lipase activity in p37 function is unclear but may involve p37-induced membrane alterations, since transfection studies have shown that deletion of the HKD motif inhibited the formation of p37-induced vesicles [11]. Whether or not ST-246 inhibits the putative phospholipase activity is currently under investigation. Taken together, our results suggest that p37-induced vesicle formation requires association of p37 with host proteins associated with LE transport vesicle biogenesis. Thus, p37 may interact with LE proteins, mimicking cellular cargo, to nucleate a membrane complex essential for the envelopment of IMV particles.

## Competing interests

All of the author(s) are employees of SIGA Technologies, Inc.

## Authors' contributions

RJ conceived the study, coordinated the research efforts, and helped write and edit the manuscript. CB conducted the buoyant density centrifugation experiments and assisted in writing and editing the manuscript. CH assisted with the immunoblots. KH, YC, and GY conducted the remaining experiments and wrote the manuscript. DH coordinated the research efforts and edited the manuscript.

## Supplementary Material

Additional file 1 **Localization of wild-type and mutant p37-GFP and CI-MPR in BSC-40 cells**. Confocal lasar scanning microscopy images showing that mutation of p37 did not affect the subcellular distribution of p37-GFP Click here for file
