# Supplementary material for: Vaccinia virus p37 interacts with host proteins associated with LE-derived transport vesicle biogenesis
Source: Virol J. 2009 Apr 28;6:44. doi: 10.1186/1743-422X-6-44 (PMC2685784; doi:10.1186/1743-422X-6-44)
Supplement: Additional file 1 — Localization of wild-type and mutant p37-GFP and CI-MPR in BSC-40 cells. Confocal lasar scanning microscopy images showing that mutation of p37 did not affect the subcellular distribution of p37-GFP [file 1743-422X-6-44-S1.ppt]

## Slide 1
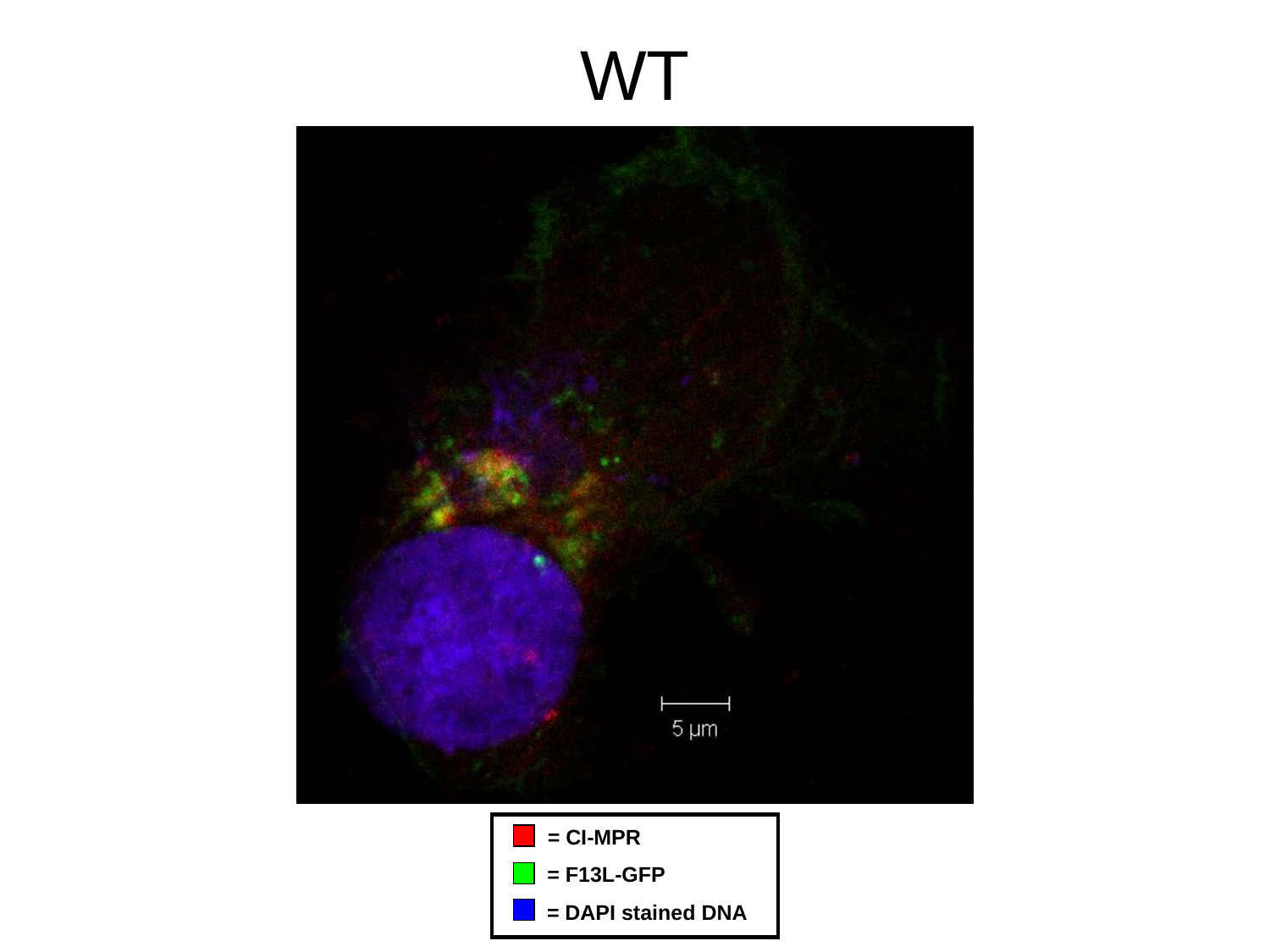

# WT
= CI-MPR
= F13L-GFP
= DAPI stained DNA

## Slide 2
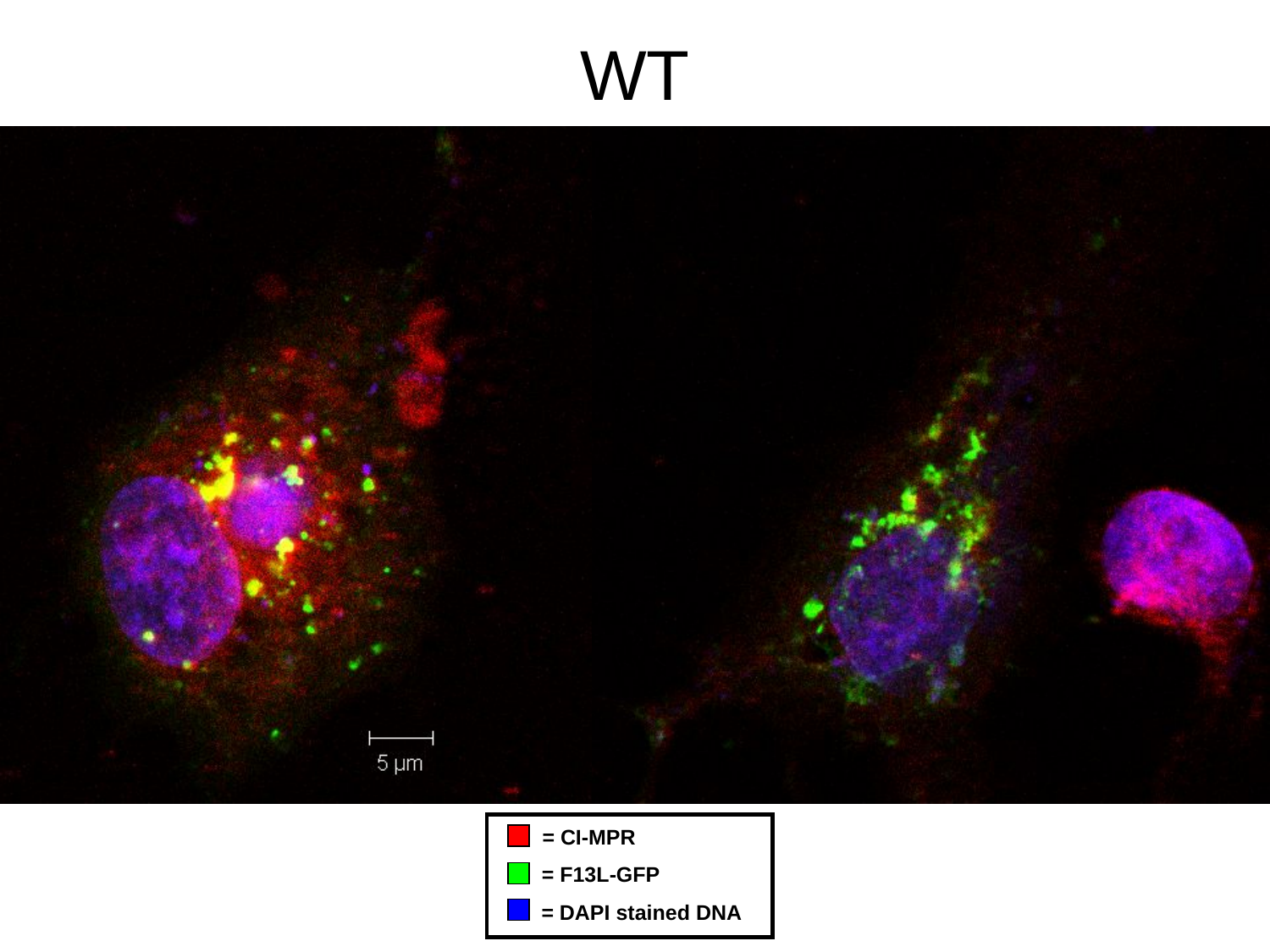

# WT
= CI-MPR
= F13L-GFP
= DAPI stained DNA

## Slide 3
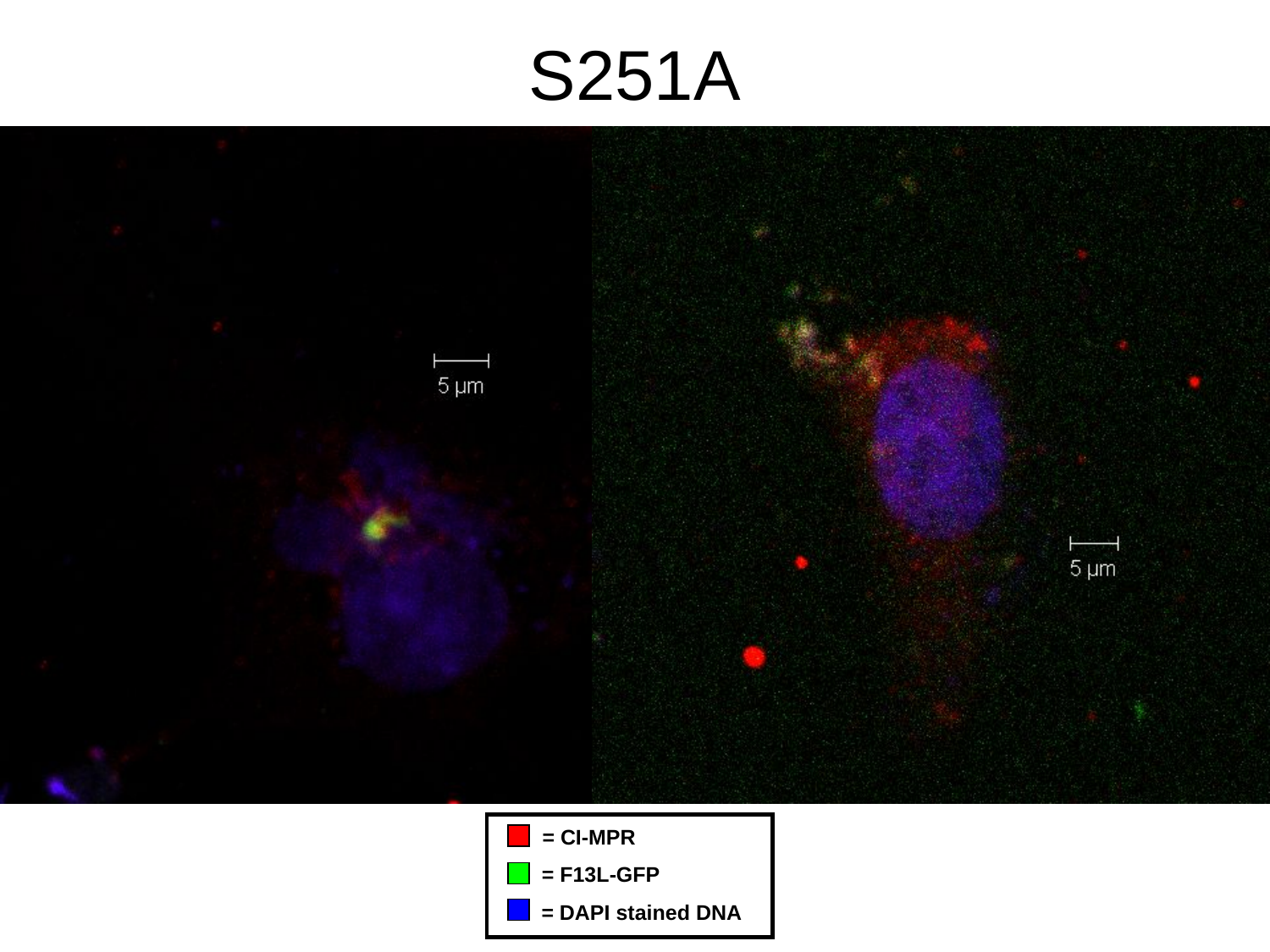

# S251A
= CI-MPR
= F13L-GFP
= DAPI stained DNA

## Slide 4
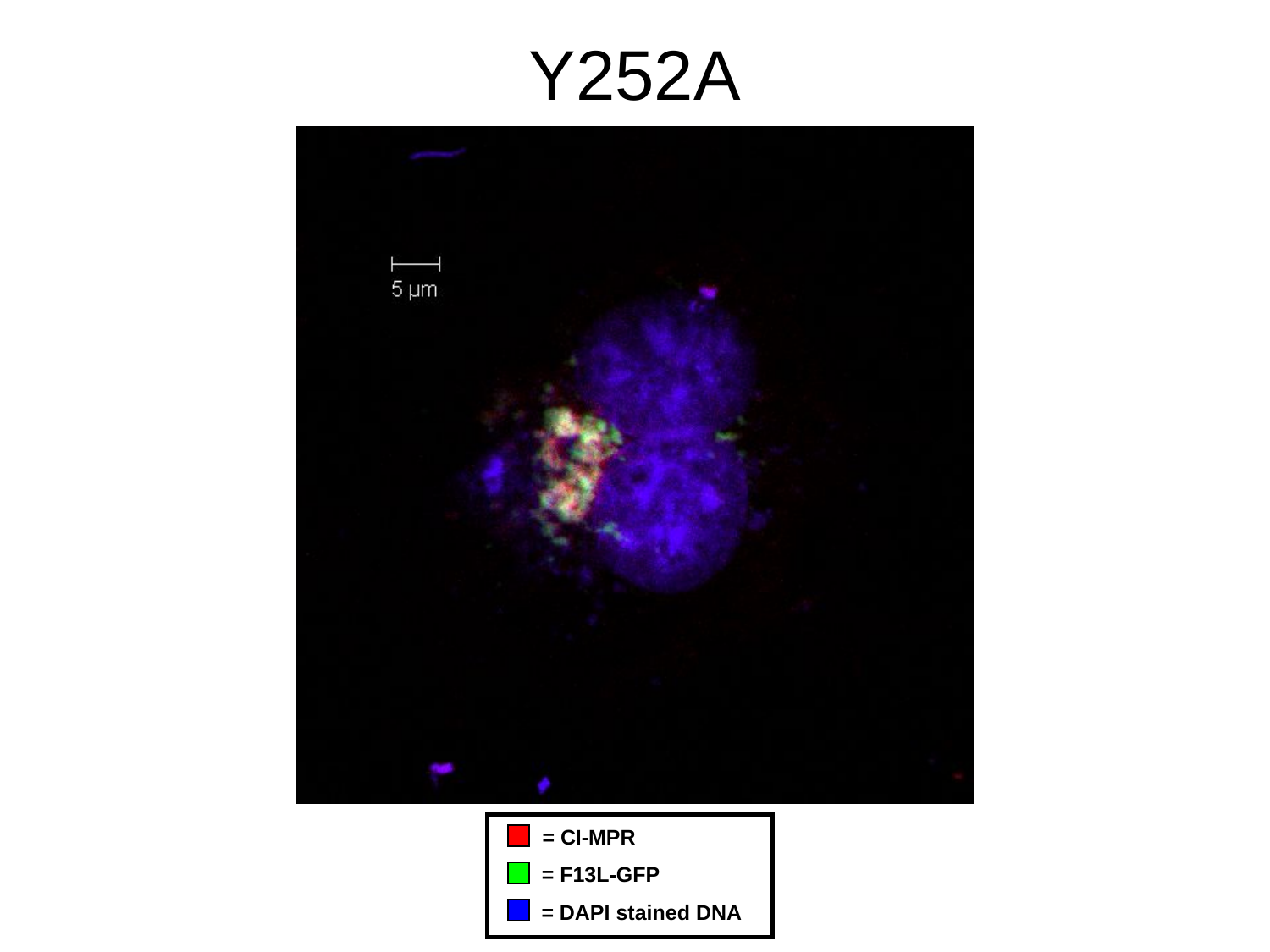

# Y252A
= CI-MPR
= F13L-GFP
= DAPI stained DNA

## Slide 5
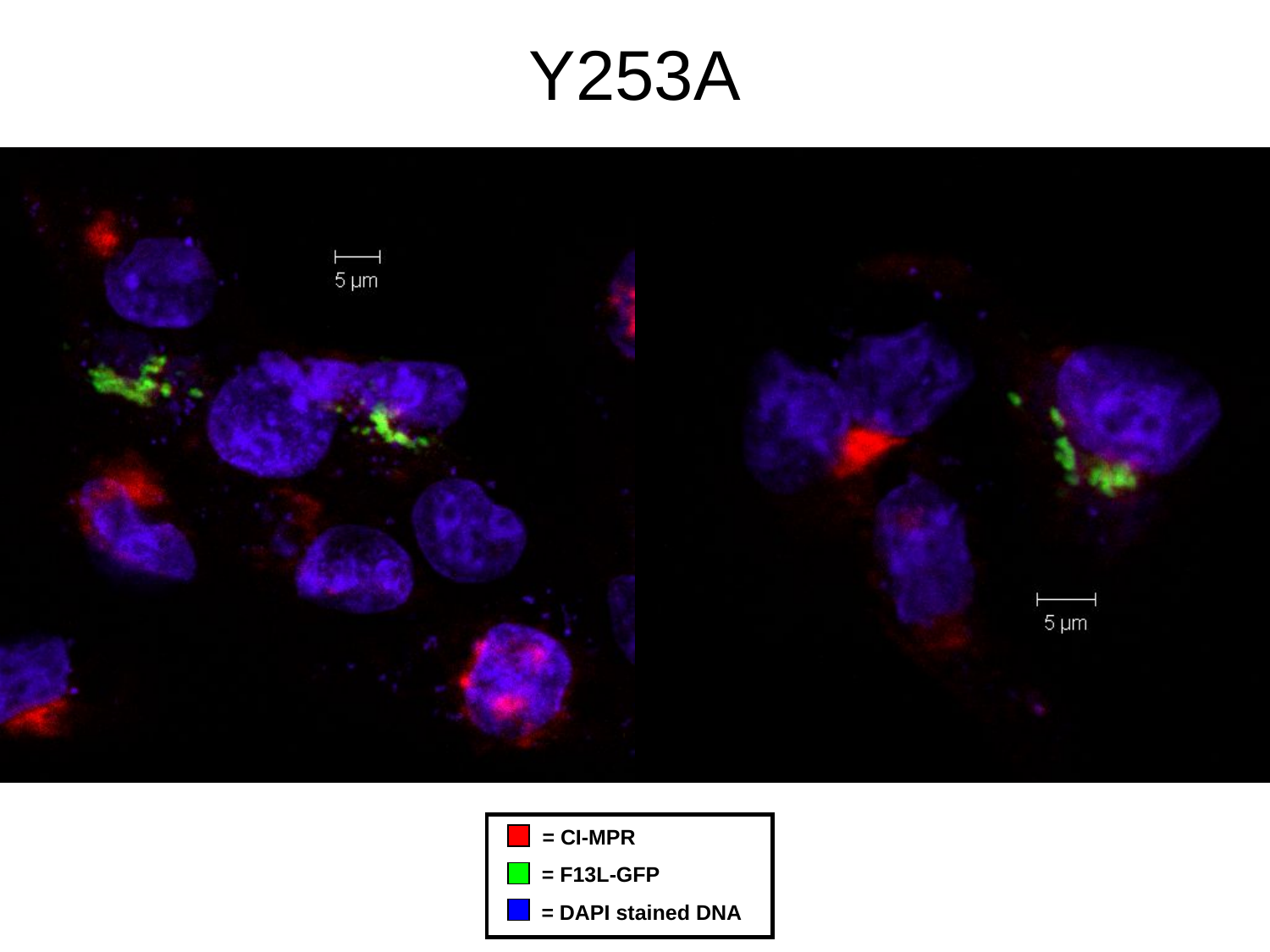

# Y253A
= CI-MPR
= F13L-GFP
= DAPI stained DNA

## Slide 6
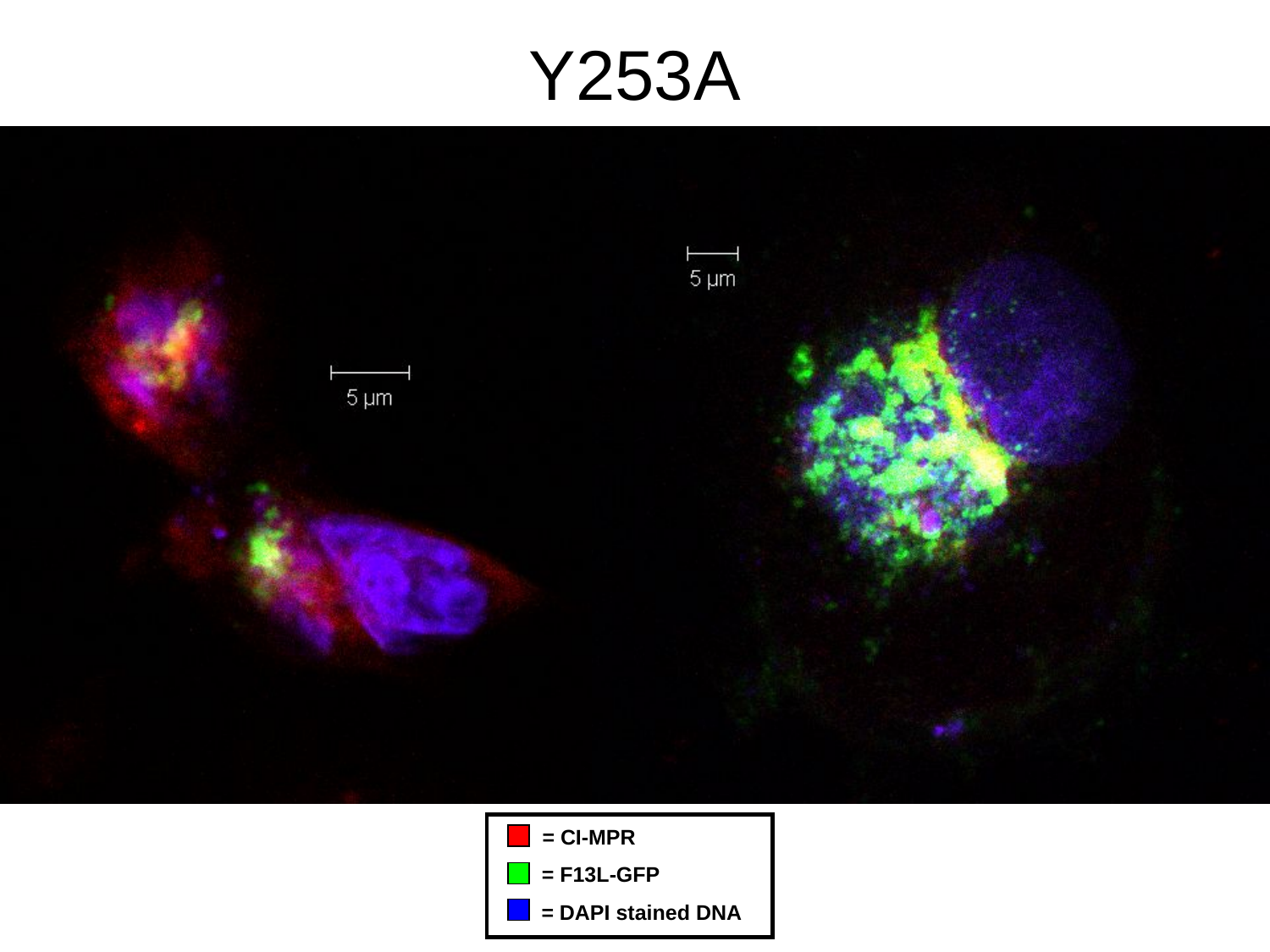

# Y253A
= CI-MPR
= F13L-GFP
= DAPI stained DNA

## Slide 7
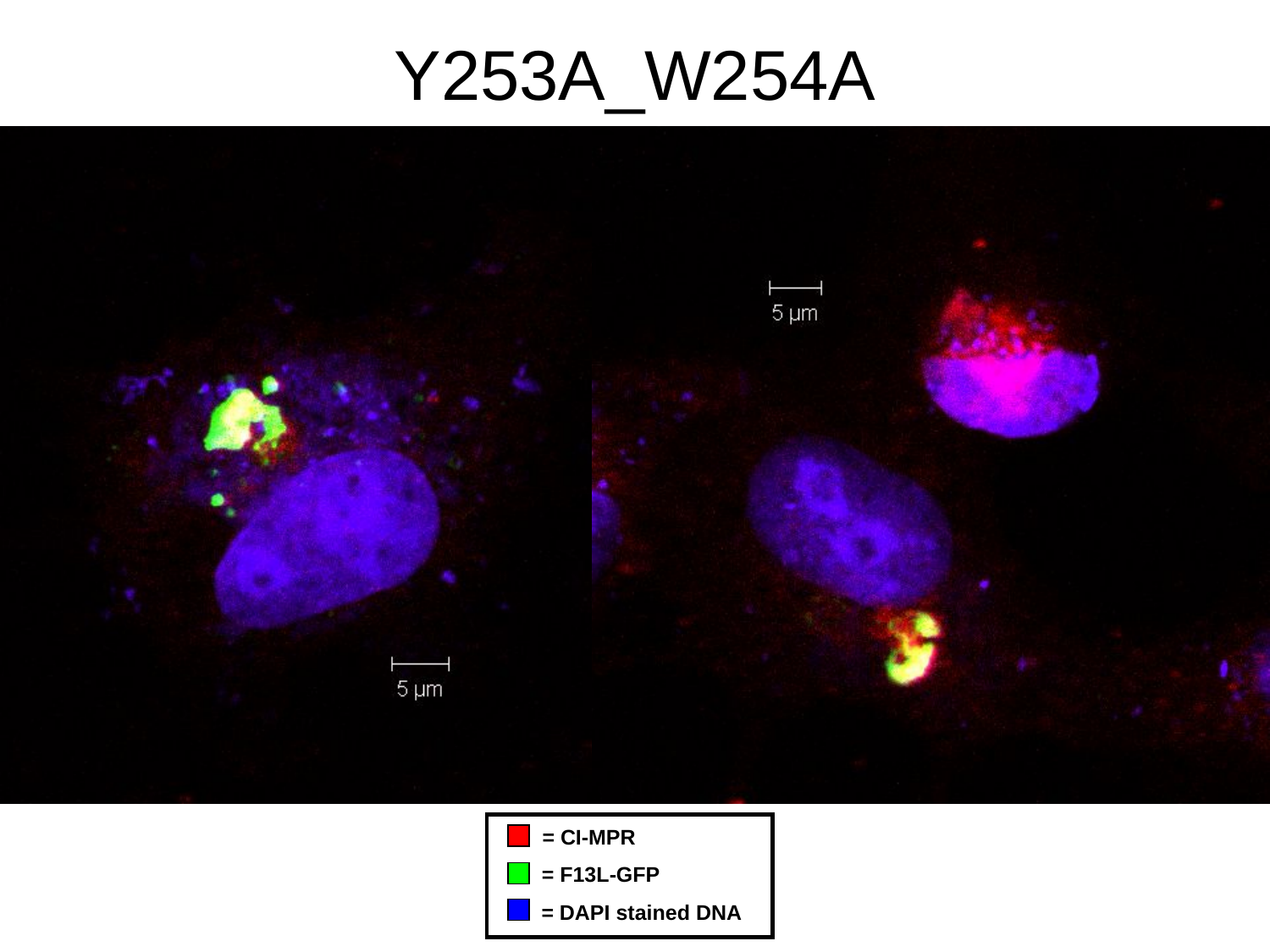

# Y253A_W254A
= CI-MPR
= F13L-GFP
= DAPI stained DNA

## Slide 8
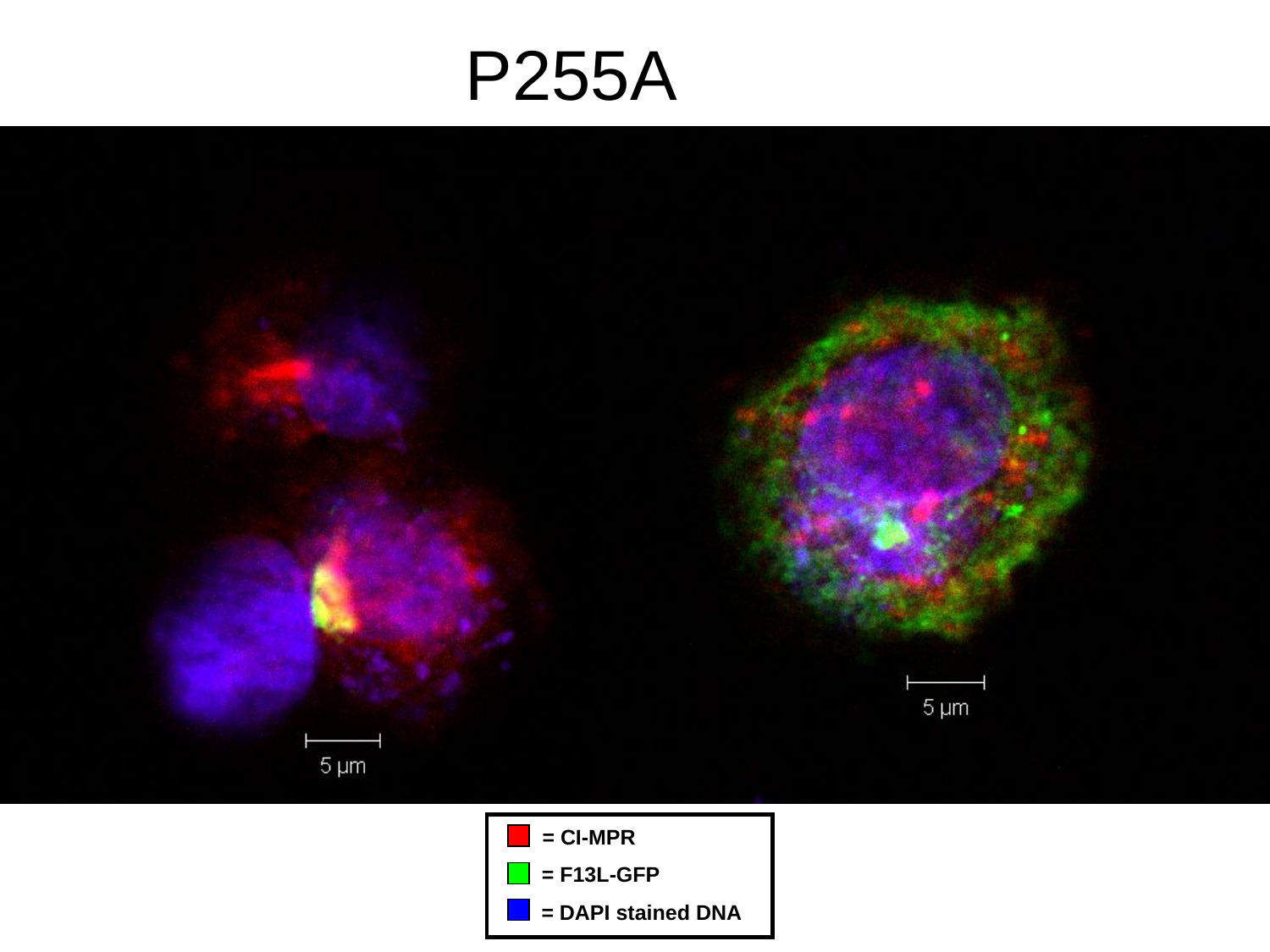

# P255A
= CI-MPR
= F13L-GFP
= DAPI stained DNA
